# Motion-Blurred Particle Image Restoration for On-Line Wear Monitoring

**DOI:** 10.3390/s150408173

**Published:** 2015-04-08

**Authors:** Yeping Peng, Tonghai Wu, Shuo Wang, Ngaiming Kwok, Zhongxiao Peng

**Affiliations:** 1 Key Laboratory of Education Ministry for Modern Design and Rotor-Bearing System, Xi’an Jiaotong University, Xi’an 710049, China; E-Mails: pyp8020@163.com (Y.P.); wstyxjtu@163.com (S.W.); 2 School of Mechanical and Manufacturing Engineering, The University of New South Wales, Sydney, NSW 2052, Australia; E-Mails: nmkwok@unsw.edu.au (N.K.); z.peng@unsw.edu.au (Z.P.)

**Keywords:** image restoration, particle separation, wear particle, on-line wear monitoring

## Abstract

On-line images of wear debris contain important information for real-time condition monitoring, and a dynamic imaging technique can eliminate particle overlaps commonly found in static images, for instance, acquired using ferrography. However, dynamic wear debris images captured in a running machine are unavoidably blurred because the particles in lubricant are in motion. Hence, it is difficult to acquire reliable images of wear debris with an adequate resolution for particle feature extraction. In order to obtain sharp wear particle images, an image processing approach is proposed. Blurred particles were firstly separated from the static background by utilizing a background subtraction method. Second, the point spread function was estimated using power cepstrum to determine the blur direction and length. Then, the Wiener filter algorithm was adopted to perform image restoration to improve the image quality. Finally, experiments were conducted with a large number of dynamic particle images to validate the effectiveness of the proposed method and the performance of the approach was also evaluated. This study provides a new practical approach to acquire clear images for on-line wear monitoring.

## 1. Introduction

Digital image processing technology is widely used in engineering applications, such as remote sensing [1,2], 3D modeling [3], object recognition [4,5], and machine condition monitoring [6,7]. In particular, to meet the on-line monitoring requirement for fault detection and diagnosis, versatile oil monitoring sensors have been developed in the past decades. There are five major groups of existing sensors classified according to their physical principles as follows [8]. Photoelectric-based sensors can provide the contour of particle projection by using a photoelectric conversion device based on shading principle. Induction sensors take advantage of the physical phenomenon that a moving wear particle would disturb a pre-set electromagnetic field to obtain the volume information of particles. Meanwhile, equivalent size, rather than a physical one, can be calculated by equating to a sphere. Electric sensors adopt a similar principle with inductive ones where the electromagnetic field is replaced with an electric field. Sensors based on ultrasonic principle, close to photoelectric one, employ the reflection of ultrasonic by solid particles to detect the object area. Imaging-based sensors are used to capture images to extract profound information of wear particles, including quantity, size, morphologies and color, for wear process and mechanism examinations.

Ferrography, the most common technique for wear particle analysis, is widely used to acquire the information of wear debris utilizing image processing [9]. An on-line visual ferrograph sensor (OLVF), which belongs to the imaging-based sensor group, was designed to capture wear debris images to extract particle features [10,11] for on-line monitoring purpose. Since the OLVF sensor system uses magnetics to attract wear debris during image acquisition, particles often appear in chained patterns. The commonly observed and reported particle overlap issue associated with the ferrograph technique makes the method not suitable for the characterization of an individual particle because it is difficult to separate the debris for diagnostic analysis. To deal with this limitation in on-line wear condition monitoring based on static wear particle images, a dynamic multi-view image acquisition system was developed for particle acquisition and feature extraction [12]. Unfortunately, dynamic wear debris images are often blurred because the particles are set in motion due to lubricant flow. This leads to severe difficulties in extracting the useful information of the wear debris. In order to alleviate the challenges in processing dynamic wear debris images, it is necessary to improve the quality of the image, among which motion-blurred image recovery is a major problem to be solved.

Relative movements between particles, lubricant and camera are the main causes of motion blur in the dynamic wear debris image. But the background of the particle image is usually regarded as static because its appearance is only affected by the lubricant. The property of the lubricant remains stable in a short duration when the machine is running, and this makes the background look similar. Thus, the dynamic particle image is categorized as a partial motion-blurred image. Despite researches on image restoration have been widely reported, most of them had focused on the whole image and partial blur issue was seldom explored.

In order to deal with the partial blur problem, three issues need to be addressed. They are partial blur detection, blur angle and length estimation, and partial deblurring. The blur regions identification is of profound importance in local blurred image processing, and there are two successful approaches reported. Chen [13] proposed an approach of directional high-frequency energy analysis to detect motion blurred regions. This method assumes that objects are in motion with a constant velocity. However, wear particle flowing in the lubricant are moving at various velocities, making it difficult to apply the directional high-frequency energy analysis approach to perform partial blur detection. The other blur detection approach is based on morphology for objects segmentation to separate blur regions [14]. Moving particles are the objects in an on-line wear debris image. But the morphology method is not sufficient for wear particle segmentation because of the influence from uneven lighting and reduction of contrast due to oil opaqueness [15]. The gradient-based object segmentation hence cannot be directly applied to effectively extract blurred particles because the gray values of the particles are close to that of the background.

With regard to blur estimation, as the degraded image is the result of the convolution between a sharp image and a point spread function (PSF), the Fourier transform based technique is one of the practical measures to restore blurred images. It is because the PSF parameters can be found from the given blurred image by further converting the image to the spectrum and cepstrum domain [16,17]. In image deblurring, many image restoration algorithms have been reported, such as Lucy-Richardson [18], least squares [19], blind deconvolution [20] and Wiener filter [2]. In the existing approaches, Wiener filter is a method with good restoration effectiveness and calculation simplicity [21]. It is also an attractive candidate for blur estimation and restoration of dynamic wear debris images in real-time wear condition monitoring.

In this work, a method is developed to restore motion-blurred wear debris images. The aim is to improve the quality of the blurred particles and segment the particles for on-line wear monitoring. This approach involves three phases. In the first stage, the static background image is sampled using the video-based debris imaging system. Particles are separated from the background based on background subtraction method [22]. In this step, the partial blur regions are detected. Second, PSF parameters including the blur direction and blur length of each blurred particle are estimated using power cepstrum. Finally, the Wiener filter algorithm is adopted to restore the segmented image based on the average PSF values to output an improved quality image.

The rest of the paper is organized as follows. In Section 2, the wear debris imaging system in on-line monitoring is introduced, as well as the characteristics of dynamic particle image. The traditional image restoration process using Wiener filter is described in Section 3. The developed approach of particle image processing is presented in Section 4. Section 5 reports experiments carried out to verify the performance of the presented method. The effective factors on the results are also discussed. Finally, conclusions are drawn in Section 6.

## 2. On-Line Wear Debris Monitoring

### 2.1. Dynamic Wear Debris Imaging System

**Figure 1:**
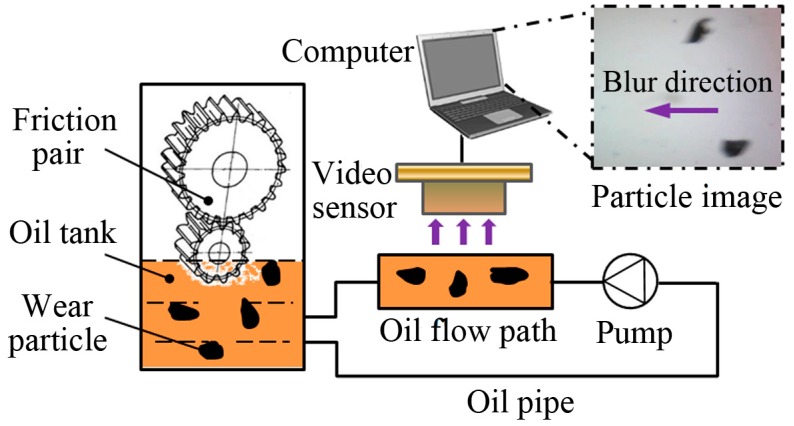
Schematic diagram of dynamic wear debris imaging system.

Figure 1 illustrates the principle of the developed on-line wear debris image acquisition system for machine condition monitoring. The recycled oil circuit was designed specifically for on-line monitoring purpose. The process of dynamic wear debris image acquisition is described as follows. Wear particles generated from a friction pair fall into an oil tank. Wear debris carried in the lubricant is firstly directed to a designed oil flow path by a digital micro pump. The pump is controlled with lower limits of 1 mL/min. Particles passing through the flow path are then imaged using a video sensor with a field of view of 1026 × 770 μm^2^. The video is captured at a rate of 15 frames per second (fps).

At the beginning of the monitoring, a no-particle image is acquired as the calibration background image to reconstruct the real-time background image by background updating. The purpose and details of background reconstruction will be described later. The reference background image and dynamic particle images are used for further wear debris analysis (WDA). Each image is of 640 × 480 pixels and is stored in the JPEG format.

### 2.2. Characteristics of Dynamic Particle Image

**Figure 2:**
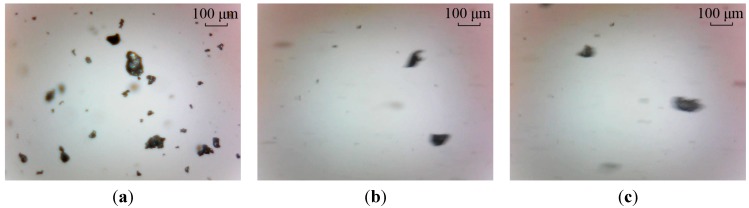
Three typical wear debris images: (**a**) static wear particle image; and (**b**,**c**) dynamic particle images.

Three sampled particle images, a static image and two dynamic images, captured with the wear debris imaging system are shown in Figure 2. These images are captured under the same focal length of the video sensor. Comparing to the static image in Figure 2a, two main characteristics of the dynamic images shown in Figure 2b,c are revealed as follows.

(1) All the dynamic images are blurred mainly causing by the movements of the wear particles. This makes it difficult to extract the effective characteristics of the wear particles. Improving the quality of the image is required to obtain the useful information of the dynamic images.
(2) The backgrounds of the two dynamic images are similar. This feature can be used in the particles separation by subtracting the background.

## 3. Related Works on Image Restoration

**Figure 3:**
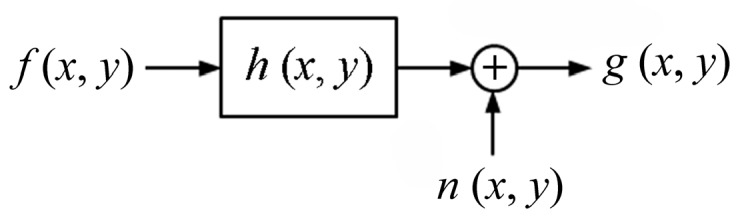
Image degradation model of motion blur [15].

A blurred image in *xy*-coordinates can be expressed with a degradation model depicted in Figure 3. As shown in the figure, a motion-blurred image *g*(*x*, *y*) is represented in the form of convolution operation between the sharp image *f*(*x*, *y*) and a degradation function *h*(*x*, *y*), and the resultant image is usually affected by additive noise *n*(*x*, *y*).

The mathematical relationship between the final blurred image *g*(*x*, *y*), the sharp image *f*(*x*, *y*), degradation function *h*(*x*, *y*) and random noise *n*(*x*, *y*) is expressed as

(1) g(x, y)=f(x, y)⋅h(x, y)+n(x, y)

where “*” denotes the convolution operator.

The blurring operation can be converted to the Fourier transform domain as

(2) G(u, v)=F(u, v)H(u, v)+N(x, y)

where *G*(*u*, *v*), *F*(*u*, *v*), *H*(*u*, *v*) and *N*(*u*, *v*) are the results of Fourier transformation of *g*(*x*, *y*), *f*(*x*, *y*), *h*(*x*, *y*) and *n*(*x*, *y*), respectively.

In order to obtain the sharp image *f*(*x*, *y*) by reversing the degradation process, the Wiener filter algorithm [23] is used to restore the particle images. The aim of image restoration is to acquire an estimated image $\tilde{f}\left(x,\;y\right)$ of the original and sharp image *f*(*x*, *y*) with a least mean square error between the two images. The mathematical expression of the Wiener filter is [23]

(3) $$\tilde{F}\left(u,\;v\right)=G\left(u,\;v\right)\cdot \frac{{\left|H\left(u,\;v\right)\right|}^{2}}{H\left(u,\;v\right)\cdot {\left|H\left(u,\;v\right)\right|}^{2}+\text{γ}}$$

where, $\tilde{F}\left(u,\;v\right)$ is the Fourier transform of $\tilde{f}\left(x,\;y\right)$; γ is the noise-to-signal power ratio of the additive noise.

The degradation function *h*(*x*, *y*) is commonly known as the point spread function. In a motion-blurred image, the PSF is affected by two factors, that are, blur length *L* and blur angle θ, which are calculated by [24]

(4) $$h\left(x,\;y\right)=\{\begin{array}{c} 1/L,\\ 0, \end{array}\;\begin{array}{c} if\;\sqrt{x^{2}+y^{2}}<L/2,\;x/y=-\tan\text{θ}\\ otherwise \end{array}$$

where, *x* and *y* are the horizontal and vertical movement directions by some distance, respectively.

Consequently, the estimated parameters, *L* and θ, are the basis for image restoration. As Equation (2) shows, the degraded image of the convolution result can be transformed to Fourier spectrum domain. Therefore, the blur information contained in the PSF parameters can be extracted by inverse Fourier transform, which is the power cepstrum. The cepstrum *C_g_*(*p*, *q*) of an image is defined as

(5) $$Cg\left(p,\;q\right)=FFT^{-1}\left\{\log\left|G\left(u,\;v\right)\right|\right\}$$

where *FFT*^−1^{·} is the inverse Fourier transformation. As can be seen, the power cepstrum is the inverse Fourier transform of the logarithm of the squared magnitude of the Fourier transform of the degraded image. Fourier spectral null is unavoidable in an image, and the logarithm of zero is negative infinite. In order to ensure the effectiveness of Equation (5), the cepstrum of an image is generally calculated using [25]

(6) $$Cg\left(p,\;q\right)=FFT^{-1}\left\{\log\left[1+\left|G\left(u,\;v\right)\right|\right]\right\}$$

**Figure 4:**
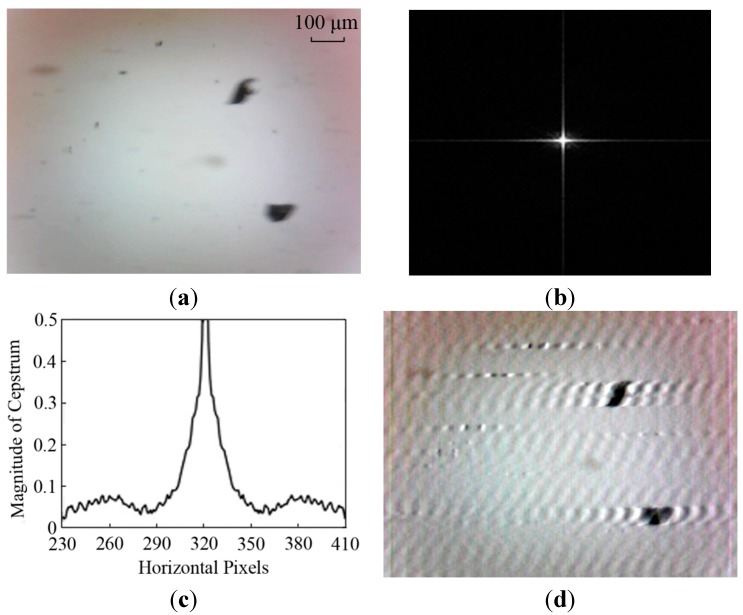
Restored results based on the whole image: (**a**) dynamic wear debris image; (**b**) power cepstrum; (**c**) cepstrum Magnitude; and (**d**) output image.

By transforming the degraded image (see Figure 4a) to the cepstrum domain as shown in Figure 4b,c, the PSF parameters, *L* and θ, are determined. Generally, the direction of motion blur is estimated by measuring the angle between any one of the parallel stripes and the horizontal axis in the cepstrum chart. From Figure 4b, parallel stripes can hardly be found. The blur angle is estimated as θ = 0° where the direction of particle motion is aligned to 0° by adjusting the camera position. In addition, there are two symmetric lowest peaks in a cepstrum magnitude curve. The distance between two peaks is equal to twice the blur length. However, the cepstrum in Figure 4c is affected by the oil contaminants in the background. The blur length in the example shown in Figure 4a is approximately determined as *L* = 41 pixels.

When the PSF parameters are estimated, the motion-blurred image can be restored based on Wiener filter method and the output image is shown in Figure 4d. It is observed that the particles become sharper but there are artifacts appearing in the background. This result caused by over-restoration of an entire image is unsatisfactory to extract accurate wear particle information.

## 4. Particle Separation for Deblurring—A New Approach

### 4.1. System Overview

**Figure 5:**
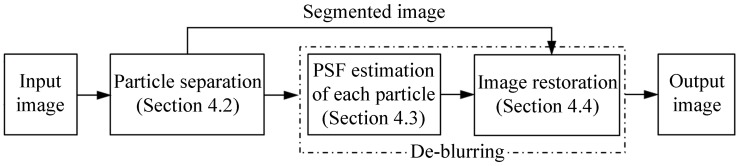
Flowchart of the proposed method.

To deal with the over-restoration problem, an approach of motion-blurred particle image processing based on particle separation is proposed and exhibited in Figure 5. A blurred particle image is given as an input; three major processing stages are then invoked.

(1) The blurred particles are separated from the background by utilizing a background subtraction method to deal with the partial blur problem (Section 4.2).
(2) The blur information including the PSF parameters of the blur angle and blur length of each particle is extracted in the cepstrum domain (Section 4.3).
(3) The segmented particle image is restored with Wiener filter algorithm based on the average PSF values to produce the final image (Section 4.4).

### 4.2. Particle Separation

Objects in an image can be classified as foreground and background. In this study, the regions of interest in the foreground of an image are wear particles. Background subtraction is a widely used approach for detecting moving objects [26,27]. The rationale in the approach is that of detecting the moving objects from the difference between the current frame (particle image) and a reference frame (background image), which is written as

(7) P[F(t)]=P[I(t)]−P[B]

where, *I*(*t*) is the wear debris image captured at the time *t*; *B* denotes the background image; *F*(*t*) is the difference image; *P* represents the pixel value.

**Figure 6:**
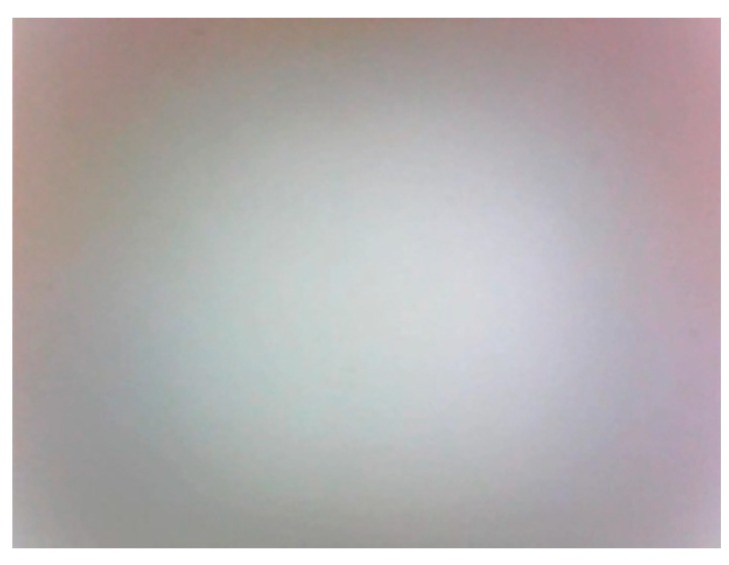
A background image.

The dynamic debris image is captured using the on-line wear debris imaging system described in Section 2.1. To obtain the background, a no-particle image was captured manually at the beginning of the monitoring process and stored in the computer as a calibration reference. Based on the reference background and a series of image frames extracted from a video, the background of a current image can be reconstructed by utilizing the Surendra background updating algorithm [28] (see Appendix A). An example of a real-time updated background image is shown in Figure 6.

**Figure 7:**
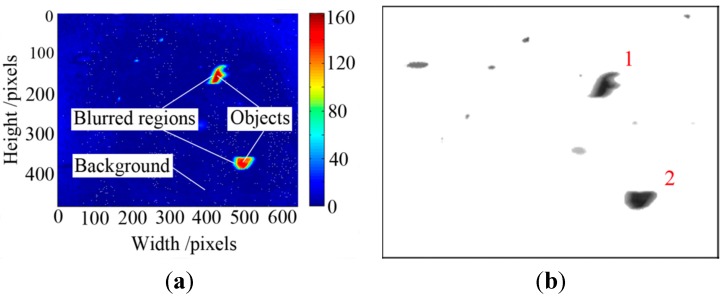
Difference and segmentation result of particle image: (**a**) pixel value of difference image; and (**b**) target debris image.

Background subtraction is based on a static background hypothesis. But in real engineering applications, some factors such as light reflections lead to background variations. The gray distribution of the difference image between wear debris image and the background are shown in Figure 7a. The smallest difference value represents the background and the highest value indicates the objects, and the others are blurred regions. Thus a threshold, *T*, is set to remove the background. We have

(8) $$P[F\left(t\right)]=\{\begin{array}{c} P[I\left(t\right)],\;\\ 255,\; \end{array}\begin{array}{c} if\;\left|P[F\left(t\right)]\right|>T\\ otherwise \end{array}$$

The threshold can be computed by using Otsu’s method [29], which is presented in Appendix B. By utilizing this algorithm, a target wear particle image was obtained based on Equation (8) and displayed in Figure 7b. The background was removed and the information of the wear particles including the motion-blurred regions was obtained.

### 4.3. PSF Estimation

**Figure 8:**
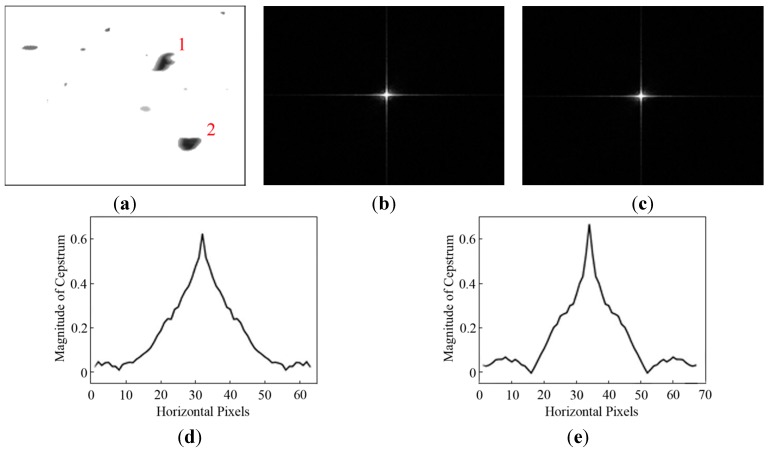
Estimated PSF of particles #1 and #2: (**a**) segmented debris image; (**b**) power cepstrum of the particle #1 with θ_1_ = 0°; (**c**) power cepstrum of the particle #2 with θ_2_ = 0°; (**d**) cepstrum magnitude of the particle #1 with *L*_1_ = 23 pixels; and (**e**) cepstrum magnitude of the particle #2 with *L*_2_ = 18 pixels.

The PSF parameters of particles were estimated with power cepstrum to determine the degraded function of the blurred particles, as described in Section 3. The cepstrums of separated wear particles #1 and #2 are shown in Figure 8. The blur angles of particles #1 and #2 are both estimated to be θ_1_ = 0° and θ_2_ = 0° from Figure 8b,c. Meanwhile, the blur lengths of the two particles are *L*_1_ = 23 pixels and *L*_2_ = 18 pixels as shown in Figure 8d,e, respectively. As can be seen, the blur length of each particle is largely different from that of the whole image. But the difference in blur lengths between the particles is small due to the short exposure interval. Hence, the average blur angle and length are adopted as the PSF values of the whole image in Figure 8, which are θ_avg_ = 0° and *L*_avg_ = 21.

### 4.4. Image Restoration Based on the Particle Separation

**Figure 9:**
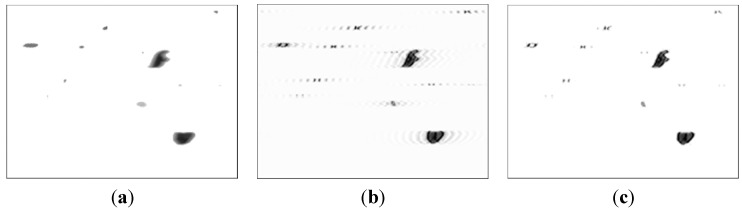
Result images during the restoration process: (**a**) original segmented image; (**b**) restored image; and (**c**) output image after morphological operation.

The segmented image in Figure 9a was restored using the Wiener filter method based on the estimated PSF parameters and the result is shown in Figure 9b. The background subtraction method was utilized again to obtain the restored particles. The artifacts appearing in the background were removed and the final image is displayed in Figure 9c. It can be seen that the particles in Figure 9c are clearer and sharper than the original ones shown in Figure 9a. The segmented particle image is appropriate for extracting the wear debris information for WDA.

## 5. Experimental Results and Discussion

To evaluate the performance of the proposed motion-blurred image restoration method, dynamic wear debris images were captured from on-line monitoring lubrication samples of a mine scraper conveyor gearbox. The video-based imaging system in Figure 1 was used to capture particle images. All the images are of 640 × 480 pixels in the JPEG format. Aiming for performance evaluations, two tests were carried out with a total of 110 images. The first test includes images restored using the existing approach on the whole image (see Section 3) and the proposed approach (Section 4), respectively. The second test is to compare the Wiener filter (WF) with other three common image restoration methods that are Lucy-Richardson (LR) [18], blind deconvolution (BD) [20] and least squares (LS) [19].

### 5.1. Comparative Test One

**Figure 10:**
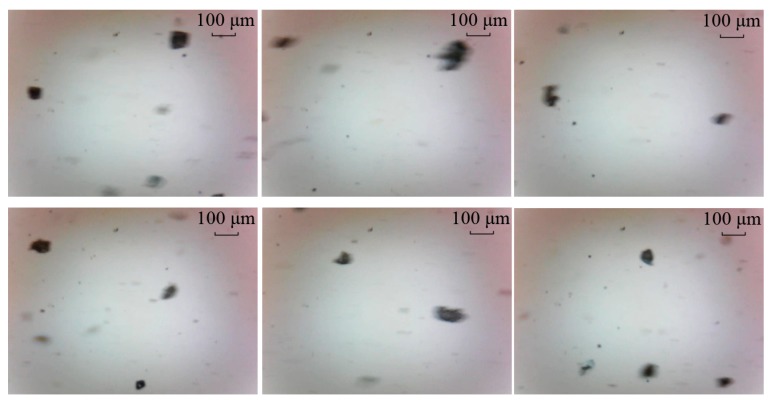
Six input particle images.

**Figure 11:**
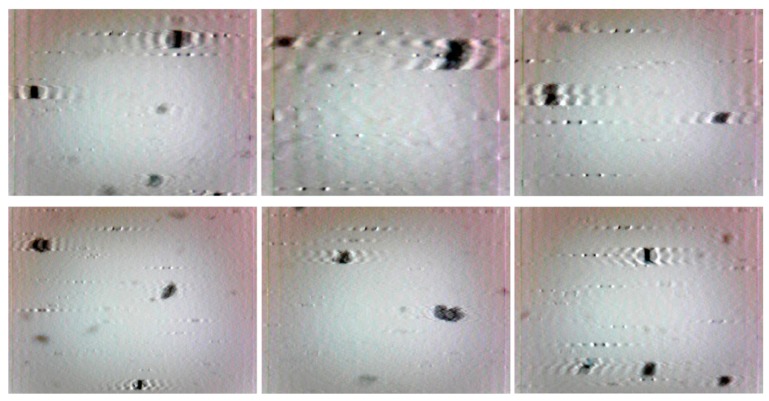
Restored results based on the whole image.

**Figure 12:**
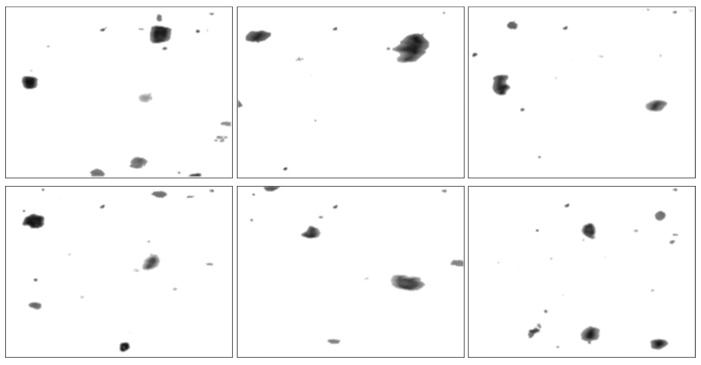
Segmented particle images using the approach presented in Section 4.

**Figure 13:**
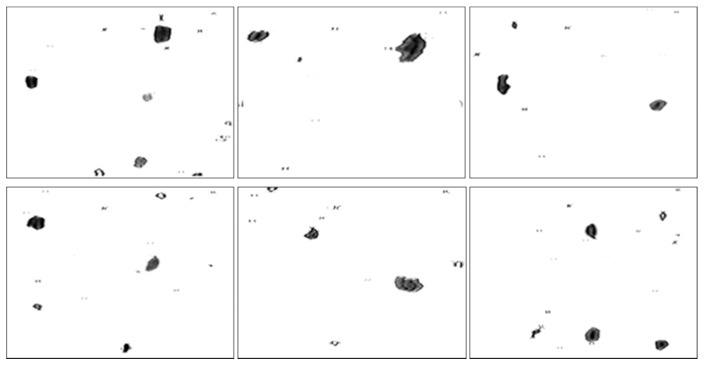
Restored images using the proposed method.

To examine the potential advantages of the proposed method, dynamic wear debris images were obtained to access the performance of the proposed local blurred particle restoration approach. Six tested images as the input are depicted in Figure 10. The processed results, using the existing image restoration method based on the whole image, are shown in Figure 11. The segmented particle images and the restored results, using the newly developed approach, are displayed in Figure 12 and Figure 13, respectively.

It can be seen from Figure 11 that most of the particles were over-restored because the estimated blur length based on the whole image was larger than that of the blurred debris. At the same time, undesirable artifacts introduced in the background make these images not desirable to do image pre-processing for debris features extraction.

In contrast, Figure 12 demonstrates that the similar background in Figure 10 was removed by utilizing the particle separation method. But the information of particles cannot be extracted directly from the segmented images because the blur regions due to the movements of particles are retained, which will affect the accuracy of wear debris feature extraction.

As shown in Figure 13, the sharpness of the particles was improved. The restored particles based on the proposed method of object separation are sharper than that based on the whole image, which is more suitable for WDA in a subjective visual assessment. In addition, the grey contrast between particles and background was also increased comparing to that in Figure 12, making it easy to extract the information of particles.

For further analysis, the proposed method was also assessed quantitatively. The purpose of motion-blurred wear debris image restoration is to improve the contrast between particles and background to extract debris features. Pixel gradient, *G*, is a sharpness measure to evaluate the object edges. It is calculated from the neighborhood pixel gradient $\tilde{G}$ as

(9) $$G=\frac{1}{N}\sum \tilde{G}\;,\;\forall uv$$

and

(10) $$\tilde{G}=\sqrt{\text{Δ}{x_{uv}}^{2}+\text{Δ}{y_{uv}}^{2}}\;u=1,\;2,\;...,\;U,\;v=1,\;2,\;...,\;V$$

where, *U* and *V* are the width and height of the image, respectively; *N* is the total number of pixels in the image and *N* = *U* × *V*; $\Delta x_{uv}=g_{uv}-g_{u+1,v}$, $\Delta y_{uv}=g_{uv}-g_{u,v+1}$ are the gradients in the horizontal and vertical directions in the image; *g* is the grey value of a pixel.

**Figure 14:**
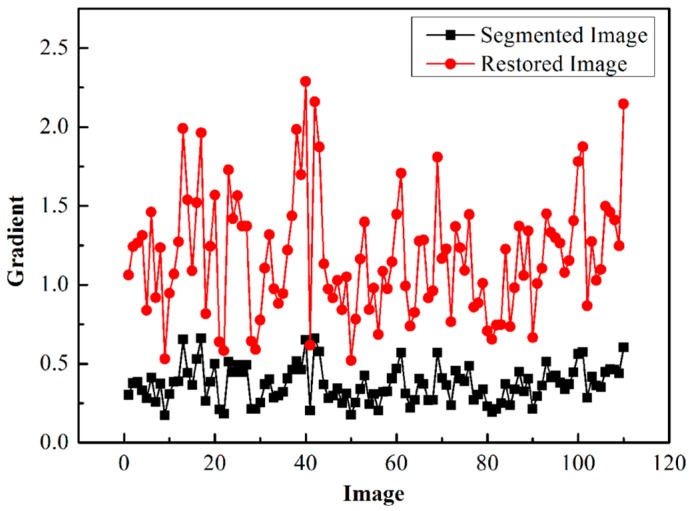
Gradients of the segmented and restored images.

All 110 images were restored using the proposed method based on the segmented images. The gradients of the original segmented images and the restored images were calculated and are displayed in Figure 14. The results show that all the gradients of the restored images in a range of [0.522, 2.288] are larger than that of the blurred images ([0.173, 0.517]). This clearly demonstrates that the restored images have much sharper edges than the original ones, proving that the proposed method of particle restoration is effective for dynamic wear debris analysis in on-line wear monitoring.

### 5.2. Comparative Test Two

**Figure 15:**
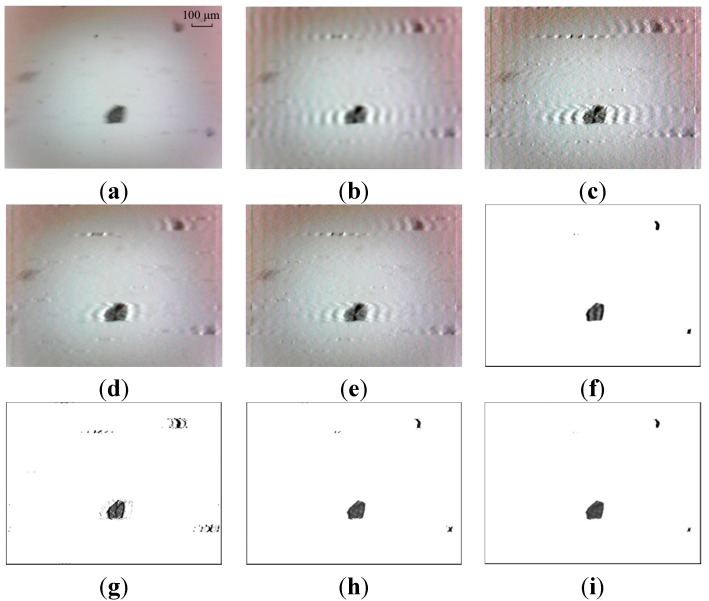
Test results—image 1: (**a**) input image; (**b**–**e**) restored images by LS, BD, LR and WF based on the whole image, respectively; and (**f**–**i**) output images by LS, BD, LR and WF based on local blurred regions, respectively.

**Figure 16:**
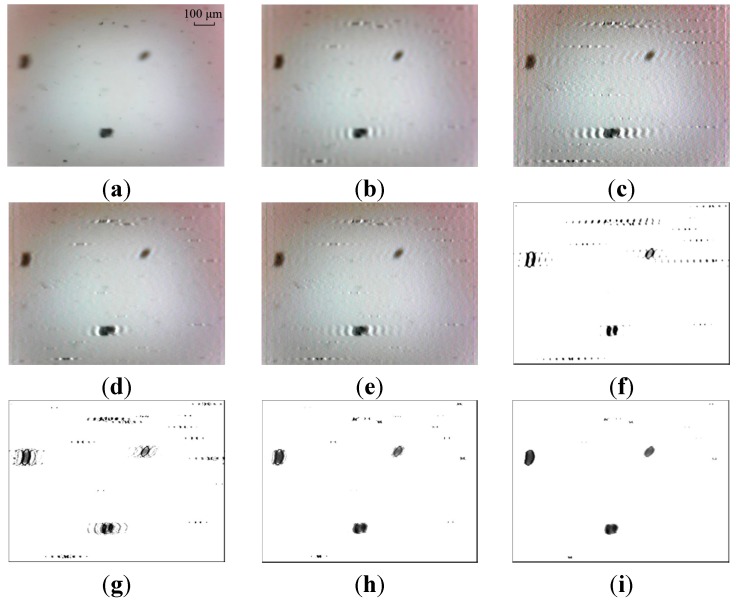
Test results—image 2: (**a**) input image; (**b**–**e**) restored images by LS, BD, LR and WF based on the whole image, respectively; and (**f**–**i**) output images by LS, BD, LR and WF based on local blurred regions, respectively.

The Wiener filter is utilized to remove the motion blur in the restoration procedure to improve the quality of images. To demonstrate the Wiener filter is more suitable for dynamic wear debris image processing, the results are compared against other three common algorithms, including Lucy-Richardson (LR), blind deconvolution (BD) and least squares (LS). Two sample images as the input images are depicted in Figure 15a and Figure 16a. The restored results using the four methods based on the whole image and the partial blurred particle regions are presented in Figure 15b–i and Figure 16b–i, respectively.

From Figure 15b–e and Figure 16b–e, it can be seen that over-restoration of the background based on the entire image makes these images not desirable to pursue image pre-processing. As shown in Figure 15f–i and Figure 16f–i, the restored results of separated wear particles are appropriate for wear debris analysis in a subjective visual assessment. The above comparisons, in addition to the comparisons made in Section 5.1, further confirm that the developed local blurred particle restoration approach works well in improving the image quality.

In Figure 15f the target particles are well restored with LS. But the result is unstable. Unwanted artifacts are sometimes introduced in the output image (see Figure 16f), which will affect the accuracy of particle extraction. This phenomenon of artifacts appearance can also be seen in the processed results by BD, as shown in Figure 15g and Figure 16g.

The LR and WF methods are better at restoring motion-blurred particle images, comparing with the methods of LS and BD, as shown in Figure 15h,i and Figure 16h,i. It is observed that the processed results are similar using the methods of LR and WF. Further comparison is described in the following.

**Figure 17:**
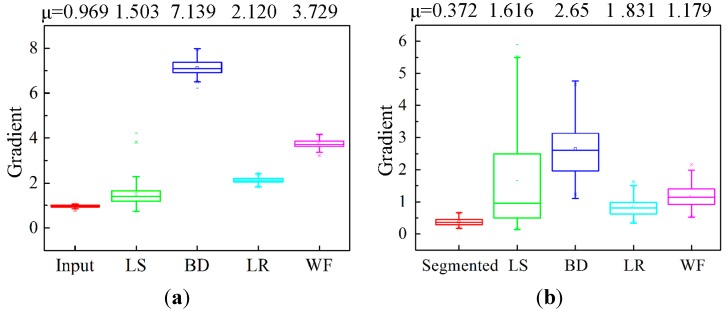
Boxplots of all 110 experimental image gradients: (**a**) gradient of the processed image based on the whole image and (**b**) gradient of the restored result based on partial blurred region.

Figure 17 shows the boxplots of all 110 experimental images gradient measures using the four common image restoration methods. In Figure 17a, the mean gradients based on the whole image are much larger than that of the input image (0.969). This is because over-restoration makes the background not smooth after image processing as mentioned above.

The mean gradients in Figure 17b show that all the gradients of the restored images by the four methods are larger than that of the blurred images. But the wide statistical gradient distribution range [0.150, 5.496] and the higher interquartile range of LS indicate that the LS method is undesirable for dynamic particle image processing. In the BD method, its mean value of 2.651 is the highest due to unnecessary artifacts appearing in the image as mentioned above, which will affect the particle features extraction. The output results of the LR and WF are better than the other two methods. The higher value of 1.179 of the Wiener filter than 0.831 of the Lucy-Richardson indicates that WF algorithm is superior to dealing with the motion-blurred problem by improving the grey contrast between particles and background.

### 5.3. Further Discussion

The above comparative analyses confirm that the proposed method of particle restoration is effective in dealing with the motion blur problem in the dynamic particle imaging system. By detecting and segmenting the partial blurred particle regions, a localized approach is, for the first time, introduced for blur removal by applying deblurring operations to individual wear particles.

Furthermore, the developed approach can be applied or further developed for other applications where dynamic images need to be captured and analyzed. For example, vehicle monitoring is important in the field of intelligent transportation. The relative movements of moving cars to static cameras cause the images blurring, making it difficult for target recognition [30]. Despite the influence of external environment, the background model of current car image can be established by real-time background updating. Hence, the proposed method can be applied to restore the partial blurred car regions to obtain high quality images. Another potential application is to use the developed method to obtain clear star maps so aircraft attitudes can be estimated [2]. It is possible to develop the object separation method to segment the blurred region for target restoration using the Wiener filter algorithm to improve the quality of the star image. In short, the approach presented in this paper is not limited to improving dynamic image quality for on-line wear debris analysis; it can be developed and applied for other applications where dynamic images are captured.

More work needs to be done to further improve the developed method. First, unstable illumination affects the segmentation threshold accuracy and consequently on the background extracted. This issue is not taken into consideration in this work because a pre-calibration is carried out before acquiring the images. In future work, we will investigate automatic illumination corrections to improve the accuracy and efficiency of the particle separation. Second, it is necessary to reduce the impacts of external noises. Effected by noises, holes within particles and artifacts may be produced in the segmented image. At present, uncorrelated noise in the background image are reduced by real-time background updating. The noise in the particle images will be eliminated by utilizing median filtering and binary morphological operations [31] in the further research of extracting particle characteristics for wear mechanism examinations.

## 6. Conclusions

In order to solve the motion-blurred problem in an on-line particle imaging system for wear debris analysis, an image restoration method was developed for improving the quality of dynamic particle images. Different to existing image restoration approaches based on an entire image, this new method identifies and separates motion-blurred particles from their background first. Based on the average PSF parameters, the segmented particles are then restored using Wiener filter algorithm. The evaluations using a large number of particle images captured under a dynamic condition in on-line wear monitoring demonstrate that the quality of the restored wear debris is significantly improved. Over-restoration of the blurred particles as well as undesirable artifacts has been avoided. This study provides a feasible deblurring method for improving motion-blurred images in on-line wear monitoring application. It is also feasible to apply or further develop this method for other applications including intelligent transportation and remote sensing where dynamic images are captured.

## Appendix

### A. Surendra Background Updating Algorithm

The algorithm [28] is used to establish the background model of dynamic wear debris images, which is described in the following steps.

(1) Set the calibration image, independently captured before the monitoring process, as original background *B*_0_ (Section 4.2).
(2) Let the first input frame *I*_0_ = *B*_0_, then calculate the difference image *D_i_* between the current *i*th frame *I_i_* and the previous frame *I_i−_*_1_ and binarized against a threshold as
  (A1) $$D_{i}\left(x,\;y\right)=\{\begin{array}{c} 1,\\ 0, \end{array}\;\begin{array}{c} \left|I_{i}\left(x,\;y\right)-I_{i-1}\left(x,\;y\right)\right|\geq T\\ \left|I_{i}\left(x,\;y\right)-I_{i-1}\left(x,\;y\right)\right|<T \end{array}\;i=1,\;2,\;\cdots ,\;MAX$$
  where, the threshold *T* usually equals 27; *MAX* is the total number of video frames.
(3) Update the background image *B_i_* based on the binary difference image *D_i_*
  (A2) $$B_{i}\left(x,\;y\right)=\{\begin{array}{c} B_{i-1}\left(x,\;y\right),\\ \alpha I_{i}\left(x,\;y\right)+\left(1-\alpha \right)B_{i-1}\left(x,\;y\right), \end{array}\;\begin{array}{c} D_{i}\left(x,\;y\right)=1\\ D_{i}\left(x,\;y\right)=0 \end{array}$$
  where *B_i_* (*x*, *y*) becomes the current background image; α is weighting factor determining the friction of current input image to the previous background used in the update. By doing this, *B_i_* (*x*, *y*) can be regarded as the real-time background image of the input *i*th frame.

### B. Otsu’s Method

Otsu’s method [29] is a clustering-based algorithm for automatic image thresholding, which is used to calculate the optimum threshold separating two classes of foreground and background. The Otsu’s method is explained below.

All the pixels, *N*, of an image are presented in *L* gray levels. The number of pixels at level *i* is denoted by *n_i_*. The probability of each gray level is computed as

(B1) $$p_{i}=n_{i}/N,\;p_{i}>0\text{ and}\sum_{i=1}^{L}p_{i}=1$$

All levels can be categorized into two classes, *C*_0_ and *C*_1_, by a threshold at level *T. T*he probabilities of class occurrence (ω_0_, ω_1_) and the class mean levels (μ_0_, μ_1_), respectively, are calculated by

(B2) $${\text{ω}}_{0}=\mathrm{Pr}(C_{0})=\sum_{i=1}^{T}p_{i}$$

(B3) $${\text{ω}}_{1}=\mathrm{Pr}(C_{1})=\sum_{i=T+1}^{L}p_{i}$$

and

(B4) $${\text{μ}}_{0}=\sum_{i=1}^{T}i\cdot \mathrm{Pr}(i|C_{0})=\sum_{i=1}^{T}ip_{i}/{\text{ω}}_{0}$$

(B5) $${\text{μ}}_{1}=\sum_{i=T+1}^{L}i\cdot \mathrm{Pr}(i|C_{1})=\sum_{i=T+1}^{L}ip_{i}/{\text{ω}}_{1}$$

The variance between class *C*_0_ and class *C*_1_ (inter-class variance) is given by

(B6) $${\text{σ}}^{2}={\text{ω}}_{0}{\left({\text{μ}}_{0}-{\text{μ}}_{t}\right)}^{2}+{\text{ω}}_{1}{\left({\text{μ}}_{1}-{\text{μ}}_{t}\right)}^{2}={\text{ω}}_{0}{\text{ω}}_{1}{\left({\text{μ}}_{1}-{\text{μ}}_{0}\right)}^{2}$$

where

(B7) $${\text{μ}}_{t}=\sum_{i=1}^{L}ip_{i}$$

is the total mean level of the original image. The higher inter-class variance, the better segmentation of background and objects can be obtained. Thus, the best threshold *T* is a threshold that makes the inter-class variance be a maximum. That is, an exhaustive search is carried for 1 ≤ *T* ≤ *L* − 1, until the inter-class variance σ^2^ is maximum.

## References

[1] Huang X., Huang B., Li H. (2009). A fast level set method for synthetic aperture radar ocean image segmentation. Sensors.

[2] Zhang W., Quan W., Guo L. (2012). Blurred star image processing for star sensors under dynamic conditions. Sensors.

[3] Alsadik B., Gerke M., Vosselman G., Daham A., Jasim L. (2014). Minimal camera networks for 3d image based modeling of cultural heritage objects. Sensors.

[4] Lee C.D., Huang H.C., Yeh H.Y. (2013). The development of sun-tracking system using image processing. Sensors.

[5] Yang J., Zhang B., Shi Y. (2012). Scattering removal for finger-vein image restoration. Sensors.

[6] Raadnui S. (2005). Wear particle analysis—Utilization of quantitative computer image analysis: A review. Tribol. Int..

[7] Yuan C., Peng Z., Yan X., Zhou X. (2008). Surface roughness evolutions in sliding wear process. Wear.

[8] Wu T., Wu H., Du Y., Peng Z. (2013). Progress and trend of sensor technology for on-line oil monitoring. Sci. China Technol. Sci..

[9] Roylance B.J. (2005). Ferrography—Then and now. Tribol. Int..

[10] Wu T., Peng Y., Wu H., Zhang X., Wang J. (2014). Full-life dynamic identification of wear state based on on-line wear debris image features. Mech. Syst. Signal Process..

[11] Wu T., Mao J., Wang J., Wu J., Xie Y. (2009). A new on-line visual ferrograph. Tribol. Trans..

[12] Dan R.M. (2013). Multi-View and Three-Dimensional (3D) Images in Wear Debris Analysis (WDA). Ph.D. Thesis.

[13] Chen X., Yang J., Wu Q., Zhao J., He X. (2012). Directional high-pass filter for blurry image analysis. Signal Process. Image Commun..

[14] Wang W., Zheng J., Zhou H. (2013). Segmenting, removing and ranking partial blur. Signal Image Video Process..

[15] Peng Y., Wu T., Wang S., Peng Z. (2015). Oxidation wear monitoring based on the color extraction of on-line wear debris. Wear.

[16] Deshpande A.M., Patnaik S. On improving accuracy of PSF estimation in spectral and cepstrum domain with morphological filtering. Proceedings of the 2012 1st International Conference on Emerging Technology Trends in Electronics, Communication and Networking (ET2ECN).

[17] Shah M.J., Dalal U.D. Hough transform and cepstrum based estimation of spatial-invariant and variant motion blur parameters. Proceedings of the 2014 International Conference on Advances in Electronics, Computers and Communications (ICAECC).

[18] Kalotra R., Sagar S.A. (2014). A review: A novel algorithm for blurred image restoration in the field of medical imaging. Int. J. Adv. Res. Comput. Commun. Eng..

[19] Stanimirović P.S., Chountasis S., Pappas D., Stojanović I. (2013). Removal of blur in images based on least squares solutions. Math. Methods Appl. Sci..

[20] Ramya S., Mercy Christial T. Restoration of blurred images using blind deconvolution algorithm. Proceedings of the 2011 International Conference on Emerging Trends in Electrical and Computer Technology (ICETECT).

[21] Qin F. Blind image restoration based on Wiener filtering and defocus point spread function estimation. Proceedings of the 2012 5th International Congress on Image and Signal Processing (CISP).

[22] Mandellos N.A., Keramitsoglou I., Kiranoudis C.T. (2011). A background subtraction algorithm for detecting and tracking vehicles. Expert Syst. Appl..

[23] Yang L., Zhnag X., Ren J. Adaptive Wiener filtering with Gaussian fitted point spread function in image restoration. Proceedings of the 2011 IEEE 2nd International Conference on Software Engineering and Service Science (ICSESS).

[24] Deshpande A.M., Patnaik S. (2014). A novel modified cepstral based technique for blind estimation of motion blur. Optik Int. J. Light Electron Opt..

[25] Wu S., Lu Z., Ong E.P., Lin W. Blind image blur identification in cepstrum domain. Proceedings of the 2007 16th International Conference on Computer Communications and Networks (ICCCN).

[26] Fernandez-Sanchez E.J., Diaz J., Ros E. (2013). Background subtraction based on color and depth using active sensors. Sensors.

[27] Lee J., Park M. (2012). An adaptive background subtraction method based on kernel density estimation. Sensors.

[28] Wang C., Jia L., Chen J. Moving object detection and tracking based on improved surendra background updating algorithm. Proceedings of the Third International Conference on Digital Image Processing (ICDIP 2011).

[29] Otsu N. (1979). A threshold selection method from gray-level histograms. IEEE Trans. Systems Man Cybern..

[30] Lin H.Y., Li K.J., Chang C.H. (2008). Vehicle speed detection from a single motion blurred image. Image Vis. Comput..

[31] Su F., Fang G., Kwok N.M. (2012). Adaptive colour feature identification in image for object tracking. Math. Probl. Eng..

